# Blogs as data: using XQuery for content evaluation

**DOI:** 10.5195/jmla.2026.2338

**Published:** 2026-04-01

**Authors:** Eli Wachter, Elizabeth A. Mullen

**Affiliations:** 1 wachter7@msu.edu, Data Visualization Librarian, Michigan State University Libraries, Michigan State University, 366 West Circle Drive (DB 24), East Lansing, MI; 2 elizabeth.mullen@nih.gov, Managing Editor of Circulating Now, National Library of Medicine, National Institutes of Health, 8600 Rockville Pike, Bethesda, MD

**Keywords:** History of Medicine, Data Visualization, Data Analysis, National Library of Medicine

## Abstract

*Circulating Now*, the history of medicine blog for the National Library of Medicine (NLM), highlights blog posts written by community contributors. To evaluate the community represented within the blog, the project team explored how XQuery, a language for querying XML data, could be utilized in developing a dataset on institutions represented in the blog. The team used ChatGPT to develop the XQuery script and processed the queries through BaseX. The resulting data was transferred to Excel where additional data elements, such as geographic location and institutional type, were manually added. From this dataset, the team created visualizations in Tableau to show the over 400 unique institutions across the world represented. These visualizations supplemented an internal report for the *Circulating Now* Editorial Board, illustrating the current engagement reach of the blog and areas for future possible collaboration.

The history of medicine blog for the National Library of Medicine (NLM), *Circulating Now*, emphasizes the role of community contributions to the shared knowledge and understanding of medical history. The blog published more than one thousand posts between 2013 and 2024, including many by guest authors, connecting the NLM collection to research at institutions around the world. To evaluate the community represented within this blog, Eli Wachter, former NLM Associate Fellow, partnered with Elizabeth Mullen, Managing Editor for *Circulating Now*, to develop a strategy to pull the names of institutions from the blog’s content, connect these institutions to their geographic location and institutional type, and create visualizations to showcase the engagement reach of *Circulating Now* for the blog’s Editorial Board.

Since *Circulating Now* is hosted on WordPress with easy access to Extensible Markup Language (XML) exports, the project team explored XQuery, a language for querying XML data, as a potential data collection strategy. BaseX, an open-source data processing engine, allowed the team to query the XML files through its robust XQuery processor. The team used ChatGPT iteratively to develop the XQuery script, providing the GenAI tool with a blank template of the XML structure, the desired keywords, and the end export goal. The final version of the XQuery script identified keywords associated with institutions (such as “university,” “library,” and “center”) and pulled five words before and after each keyword to isolate the institution’s name.

Once the XQuery script was executed, the resulting data was transferred to Excel. Unnecessary words were removed manually, leaving only the institution names. Geographic information, such as city, state, and country, were added manually to the spreadsheet. For institutions that had multiple locations, and the exact location was not mentioned in the blog, the team defaulted to the main headquarters location for the institution. Institutional type (e.g., academic, library, museum, or professional organization) was assigned by keywords within the institution’s name and manually reviewed.

Importing the cleaned data into Tableau, the team created visualizations that effectively represented the institutions NLM connected with through the blog. The data included over 400 unique institutions, with high representation on the U.S. East Coast and large cities like Washington D.C., New York, and Chicago. Areas for growth included U.S. Mountain states (Idaho, Montana, Nevada, New Mexico, Utah, and Wyoming) and countries outside of North America and Western Europe. Two-thirds of represented institutions were academic, suggesting opportunities for increased direct collaboration with libraries and museums, ensuring their names are included in the post. These visualizations supplemented an internal report for the *Circulating Now* Editorial Board. As conveyed in a July 24, 2025, *Circulating Now* post authored by Wachter and entitled “Circulating Now as Data: Community Representation,” the project as a whole demonstrates the potential of using XML as a dataset, offering insights to inform future blog content, outreach, and editorial decisions.

